# By the Way

**Published:** 1913-05-17

**Authors:** 


### BY THE WAY.

### CORRECT YOUR PROOFS.

The necessity for properly correcting proofs is shown by a case reported in *Paris Médical*. A certain workman came home in an intoxicated condition and told his wife to go to the chemist and fetch something to make him sober. The wife, after consulting a popular book of medicine, discovered the following prescription: "Water 100 grammes, peppermint water 15 grammes, ammonia 15 grammes. F. M. to be taken in two or three doses," which she proceeded to transcribe, and then took it to the chemist to be made up. The chemist delivered the mixture according to the formula given, the patient took it, and at once died. Instead of 15 grammes of ammonia the proper dose should have been 15 drops. This last was the dose printed in the original edition of the work in question published at Ghent, but in the Paris reprint possessed by the woman the dose was given as grammes. Both the medical writer and the chemist were prosecuted, the former for homicide through negligence, the latter on a similar charge and, in addition, for having delivered a medicine without a proper medical prescription. The tribunal held that both prisoners were guilty, and condemned the doctor, "who has not supervised the impression of his book," to three months' imprisonment with delay, and 100 francs fine, the chemist to a similar fine and to one month's imprisonment with delay. The wife obtained 1,000 francs damages and a yearly pension of 300 francs, and, in addition, each child was ordered to receive a yearly pension of 300 francs until of age. It seems to us that the only fitting ending to such an extraordinary verdict would have been for the woman to have been convicted also, fined and imprisoned for negligence and the illegal practice of medicine! For had she done what her husband told her in the first instance and gone straight to the chemist, the tragedy would never have occurred.

